# Local Ancestry Inference in Large Pedigrees

**DOI:** 10.1038/s41598-019-57039-w

**Published:** 2020-01-13

**Authors:** Heming Wang, Tamar Sofer, Xiang Zhang, Robert C. Elston, Susan Redline, Xiaofeng Zhu

**Affiliations:** 1 Division of Sleep and Circadian Disorders, Brigham and Women’s Hospital and Harvard Medical School, Boston, MA USA; 2 Program in Medical and Population Genetics, Broad Institute, Cambridge, MA USA; 3 Department of Population and Quantitative Health Sciences, Case Western Reserve University, Cleveland, OH USA; 4 College of Information Sciences and Technology, Pennsylvania State University, University Park, PA USA; 5 Department of Sleep Medicine, Beth Israel Deaconess Medical Center, Boston, MA USA

**Keywords:** Software, Statistical methods

## Abstract

Local ancestry, defined as the genetic ancestry at a genomic location of an admixed individual, is widely used as a genetic marker in genetic association and evolutionary genetics studies. Many methods have been developed to infer the local ancestries in a set of unrelated individuals, a few of them have been extended to small nuclear families, but none can be applied to large (e.g. three-generation) pedigrees. In this study, we developed a method, FamANC, that can improve the accuracy of local ancestry inference in large pedigrees by: (1) using an existing algorithm to infer local ancestries for all individuals in a family, assuming (contrary to fact) they are unrelated, and (2) improving its accuracy by correcting inference errors using pedigree structure. Applied on African-American pedigrees from the Cleveland Family Study, FamANC was able to correct all identified Mendelian errors and most of double crossovers.

## Introduction

There has been an increasing interest in studying the ancestral spectrum of admixed individuals, such as African Americans and Latino Americans^1–3^. Investigating the different ancestral proportions across an individual’s genome, i.e. the local ancestries, is useful for estimating population-specific genetic effects via admixture mapping studies^4–6^, capturing natural selection signals^1,2,7–9^, understanding population migration history^10^, and correcting for local population structure in genome-wide association studies^11,12^.

Many methods have been developed to infer the local ancestries in unrelated admixed individuals from a given study sample using dense genotype data. In particular, Hidden Markov Model (HMM)-based methods, including SABER+^13^, HAPMIX^14^, LAMP-LD^15^, and PCAdmix^3^, are widely used because of their high accuracy and resolution. In brief, these methods model the observed genotypes of admixed individuals conditioning on the hidden states of their ancestral reference alleles or haplotypes, which are assumed to follow a Markov process. All the above methods can be applied to phased haplotypes or diploid genotypes using a joint HMM applied to the two haplotypes in an admixed individual.

Family data have multiple advantages in genetic studies compared to population-based data, both by increasing the statistical power to identify risk variants through better control of environmental confounding effects and by more precise modeling of heritability. Genomics data in large admixed pedigrees (e.g. the Cleveland Family Study^16^ [CFS] and the San Antonio Family Heart Study^17^ [SAFHS]) are available for family-based admixture mapping and association analyses. However, existing local ancestry inference methods for family data are limited. LAMP-HAP^15^ and PCAdmix^3^ were extended to small nuclear families by fitting joint HMMs on shared haplotypes among family members. For example, in a parent-offspring pair, the parent and child share one common haplotype. The family-wise local ancestries can be estimated from a joint HMM of the three independent haplotypes. In a parent-offspring trio, the child inherited one haplotype from each of the parents. The family-wise local ancestries can be estimated from the joint HMM of the four independent haplotypes. However, this design requires a complex computationally intensive process to phase the family members. The model complexity increases quadratically with the number of founders. Therefore, it can be hard to apply to large pedigrees. The common approach for inferring local ancestries in large pedigrees is currently to incorrectly assume individuals are unrelated. This approach may result in multiple Mendelian errors that violate the assumption of family-based genetic analyses.

In this study, we developed a method which estimates local ancestries in large pedigrees by: (1) using existing software (e.g. SABER+ and HAPMIX) to infer local ancestries for all individuals in a family, temporarily assuming they are unrelated, and then (2) using FamANC to improve the local ancestry inference accuracy by using the known pedigree structure to correct inference errors

## Methods

**Figure 1:**
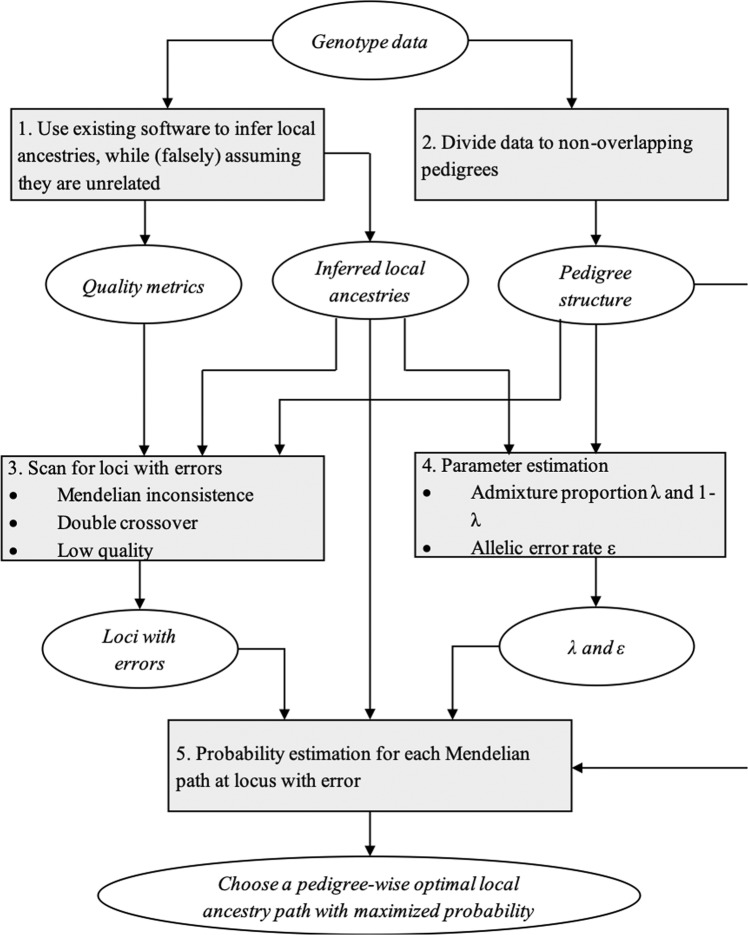
FamANC algorithm flow in a two-way admixed population.

FamANC was developed for admixed pedigrees with two genetic ancestors, such as African-Americans, who were admixed from West-African and European ancestral populations. The general flow of the FamANC algorithm is described in Fig. 1.

### Notation

Let *τ* be the number of generations since admixture occurred. Without loss of generality, we use 1 for an African (true/inferred) allele and 0 for a European (true/inferred) allele at locus *t* of individual *i*. Let *λ* and 1 − *λ* be the admixture proportions of African and European ancestry in the population. In a pedigree of *n* individuals and *L* dense markers on a chromosome, let ${X}_{t}=({X}_{1,t},{X}_{2,t},\ldots ,{X}_{n,t})$ be the true and ${Y}_{t}=({Y}_{1,t},{Y}_{2,t},\ldots ,{Y}_{n,t})$ be the inferred (using existing software) diploid local ancestries at the *t*^*th*^ locus; ${A}_{i,t}^{[1]}$ and ${A}_{i,t}^{[2]}$ be the two ancestry alleles of ${X}_{i,t}$, and ${B}_{i,t}^{[1]}$ and ${B}_{i,t}^{[2]}$ be the two alleles of ${Y}_{i,t}$, so that ${X}_{i,t}={A}_{i,t}^{[1]}+{A}_{i,t}^{[2]}$ and ${Y}_{i,t}={B}_{i,t}^{[1]}+{B}_{i,t}^{[2]}$. ${A}_{i,t},{B}_{i,t} \sim Binom(1,\lambda )$ and ${X}_{i,t},{Y}_{i,t} \sim Binom(2,\lambda )$. Let *Ped* be the set of all possible *X*_*t*_ at any *t* in a pedigree satisfying Mendelian inheritance. For example, in a nuclear family with two parents and one offspring (n = 3), the set of all possible ${X}_{t}=({X}_{1,t},{X}_{2,t},{X}_{3,t})$ corresponding to (Father, Mother, Child) is *Ped* = {(0, 0, 0), (0, 1, 0), (0, 1, 1), (0, 2, 1), (1, 0, 0), (1, 0, 1), (1, 1, 0), (1, 1, 1), (1, 1, 2), (1, 2, 1), (1, 2, 2), (2, 0, 1), (2, 1, 1), (2, 1, 2), (2, 2, 2)}.

In this study, the accuracy of the local ancestry inference is evaluated by the dosage error rate, defined as the average difference between the inferred and true local ancestries across all individuals and all loci in a given sample:

$$err=\frac{{\sum }_{i=1}^{n}{\sum }_{t=1}^{L}|{Y}_{i,t}-{X}_{i,t}\,|}{n\cdot L}$$

Let *ε* be the average allelic local ancestry inference error rate,

1 $$P({B}_{i,t}=l|{A}_{i,t}=k)=\{\begin{array}{ll}1-\varepsilon \, & l=k\\ \varepsilon \, & l\ne k\end{array},\,$$

where $l,k\,{\epsilon }\{0,1\}$. Under this model, we can compute the inference probabilities of *T*_*i,t*_ given the true local ancestry *X*_*i,t*_ as shown in Supplementary Table 1, and then the dosage error rate is estimated in terms of *ε* and *λ* as follows:

2 $$err=2\varepsilon (1-\varepsilon )+{\varepsilon }^{2}[{\lambda }^{2}+{(1-\lambda )}^{2}]$$

### Local ancestry inference error detection

After applying existing software, FamANC firstly scans local ancestry errors that arise from: (1) Mendelian inconsistencies identified from the pedigree structure; (2) double crossovers occurring within 2 cM, described as follows. We assume that the number of crossover events *R* in an interval of *d* Morgans follows a Poisson distribution, $$R \sim Pois(-\,d\tau )$$, where *τ* is the number of generations since original admixture. Letting *τ* = 8, the average number of generations since admixture in African-Americans, in a region of 2 cM the probability of observing two or more recombination events is low: $${\rm{P}}({\rm{R}}\ge 2)=1-{\rm{P}}({\rm{R}}=0)\,$$$$-{\rm{P}}({\rm{R}}=1)=0.0066$$. Therefore, we treat double crossovers within 2 cM as errors. We screen and take care of loci presenting either of these two types of errors or with low local ancestry estimation quality (<90%) based on output of existing software.

### Statistical model

FamANC corrects identified local ancestry errors using the known pedigree structure. Suppose that local ancestry is inferred at loci $t=1,\ldots .,L$, where the loci are ordered but are not necessarily adjacent. For any estimated local ancestry *Y*_*t*_ at position $t=2,\ldots ,n-1$, we correct potential error by borrowing information from the true local ancestries ${Y}_{t-1}$ and ${Y}_{t+1}$, which are assumed to be inferred without errors so that ${X}_{t-1}={Y}_{t-1}$ and ${X}_{t+1}={Y}_{t+1}$, using the following probability model:

3 $$P({X}_{t}|{Y}_{t},{X}_{t-1},{X}_{t+1})={{\rm{C}}}_{t}P({X}_{t},{Y}_{t},{X}_{t-1},{X}_{t+1}\,),$$

where ${{\rm{C}}}_{t}=\frac{1}{P({Y}_{t},{X}_{t-1},{X}_{t+1})}=\frac{1}{P({Y}_{t-1},{Y}_{t},{Y}_{t+1})}$ is a constant. Therefore, we want to identify the optimal ${X}_{t}\in Ped$, having the largest joint probability $P({X}_{t},{Y}_{t},{X}_{t-1},{X}_{t+1}\,)$. We assume that ${X}_{t-1},{X}_{t}$, and ${X}_{t+1}$ satisfy the Markov property: $P({X}_{t}|{X}_{1},\ldots ,{X}_{t-1})=P({X}_{t}|{X}_{t-1}\,)$. $P({X}_{t-1})$ is a constant.

4 $$\begin{array}{rcl}{\hat{X}}_{t} & = & argma{x}_{{X}_{t}\in Ped}P({X}_{t},{Y}_{t},{X}_{t-1},{X}_{t+1})\\  & = & argma{x}_{{X}_{t}\in Ped}P({Y}_{t}|{X}_{t},{X}_{t-1},{X}_{t+1})P({X}_{t+1}|{X}_{t},{X}_{t-1})P({X}_{t}|{X}_{t-1})P({X}_{t-1})\\  & = & argma{x}_{{X}_{t}\in Ped}P({Y}_{t}|{X}_{t})P({X}_{t+1}|{X}_{t})P({X}_{t}|{X}_{t-1}).\end{array}$$

If an error is observed at the first locus of a chromosome, i.e. when *t* = 1, then

$$P({X}_{1},{Y}_{1},{X}_{2})=P({Y}_{1}|{X}_{1})P({X}_{2}|{X}_{1}).$$

If an error occurs at the last locus of a chromosome, i.e. when *t* = *L*, then,

$$P({X}_{L},{Y}_{L},{X}_{L-1})=P({Y}_{L}|{X}_{L-1})P({X}_{L}|{X}_{L-1}\,).$$

Notably, the accuracy of correcting the inference errors for loci at the boundaries of a chromosome will be worse compared with the interior, because we can collect less information at the boundaries.

The joint probability in Eq. (4) can be decomposed into two parts: the inference probability $P({Y}_{t}|{X}_{t})$ and the transition probability $P({X}_{t+1}|{X}_{t})$. We first estimate the transition probability. We assume that the distance between *X*_*t*_ and ${X}_{t+1}$ is small enough that, in a family member *i*, the status of ${X}_{i,t+1}$ depends on ${X}_{i,t}$ but not on other family members. Therefore, the joint transition probability in a pedigree can be written as the product of the individual transition probabilities:

5 $$\begin{array}{rcl}P({X}_{t+1}|{X}_{t}) & = & P({X}_{1,t+1},{X}_{2,t+1},\ldots ,{X}_{n,t+1}|{X}_{1,t},{X}_{2,t},\ldots ,{X}_{n,t})\\  & = & \prod _{i}\,P({X}_{i,t+1}|{X}_{1,t},{X}_{2,t},\ldots ,{X}_{n,t})\\  & = & \prod _{i}\,P({X}_{i,t+1}|{X}_{i,t}).\end{array}$$

The transition probabilities are estimated from the recombination events, which are the same as those used in HMMs^13,18^. For haploid data,

6 $${p}_{kl}=P({A}_{h,t}=l|{A}_{h,t-1}=k)=\{\begin{array}{c}\exp (-\,{d}_{t}\tau )+\lambda [1-\exp (-\,{d}_{t}\tau )]\\ \lambda [1-\exp (-\,{d}_{t}\tau )]\end{array}\begin{array}{c}l=k\\ otherwise\end{array},$$

where $l,k\,{\epsilon }(0,1)$ corresponding to European ancestry and African ancestry in African Americans; *τ* is the number of generations since admixture occurred; and *d*_*t*_ is the genetic distance (in Morgans) between the *t*^th^ and *t* + *1*^th^ loci, which is usually small enough that for current GWAS array data at most one recombination event can occur. The diploid transition probability matrix is thus

We next estimate the inference probability $\,P({Y}_{t}|{X}_{t})$, which can be written as the product of the individual inference probabilities:

7 $$\begin{array}{rcl}P({Y}_{t}|{X}_{t}) & = & P({Y}_{1,t},{Y}_{2,t},\ldots ,{Y}_{n,t}|{X}_{1,t},{X}_{2,t},\ldots ,{X}_{n,t})\\  & = & \prod _{i}\,P({Y}_{i,t}|{X}_{1,t},{X}_{2,t},\ldots ,{X}_{n,t})\\  & = & \prod _{i}\,P({Y}_{i,t}|{X}_{i,t}).\end{array}$$

$P({Y}_{i,t}|{X}_{i,t})$ is given in Supplementary Table 1 with parameters *ε* and *λ*.

### Parameter estimation

λ is estimated as the average African ancestry proportion of an individual in the admixed population. Next, we estimate *ε* from the local inferred ancestries using existing software that incorrectly assumes all individuals are unrelated. Here we modified the method of estimating genotyping errors from Mendelian inconsistency in nuclear families proposed by Saunders *et al*.^19^. Some of the local ancestry inference errors will lead to Mendelian errors and others will not. From the observed number of Mendelian errors in the data, we can estimate *ε*. We first divide a large pedigree into smaller non-overlapping nuclear families. For simplicity, we assume only one member in a nuclear family can have an error at a particular position. We list all possible patterns of true and inferred local ancestries in the family members and count the number of Mendelian errors for each corresponding pattern (Supplementary Appendix 1, Supplementary Tables 2–7). Let *N*_*ME*_ be the number of Mendelian errors in a nuclear family. For a nuclear family with two parents and *m* children,

8 $$\begin{array}{c}{N}_{ME}=\\ {\varepsilon }^{2}\{2-m+[4m-12+{(\frac{1}{2})}^{m-2}]\lambda (1-\lambda )-[6m-16{(\frac{3}{4})}^{m}+{(\frac{1}{2})}^{m-3}]{\lambda }^{2}{(1-\lambda )}^{2}\}\\ +\varepsilon \{2m+[8-6m-{(\frac{1}{2})}^{m-3}]\lambda (1-\lambda )+[4m-16{(\frac{3}{4})}^{m}+{(\frac{1}{2})}^{m-4}]{\lambda }^{2}{(1-\lambda )}^{2}\}.\end{array}$$

For a nuclear family with one parent and m children,

9 $$\begin{array}{rcl}{N}_{ME} & = & 2\varepsilon (1-\varepsilon )\lambda (1-\lambda )\{2-{(1-\frac{1}{2}\lambda )}^{m}-{(\frac{1}{2}+\frac{1}{2}\lambda )}^{m}+\frac{1}{2}m\}\\  &  & +\,{\varepsilon }^{2}\{\lambda [1+\lambda -{(1-\lambda )}^{m}]+{(1-\lambda )}^{2}(2-\lambda +{\lambda }^{m})\}.\end{array}$$

The mathematical details are shown in Supplementary Appendix 1. With *N*_*ME*_ observed from the data and *λ* assumed known, we can estimate *ε* by solving Eqs. (8) and (9).

### Simulation

**Figure 2:**
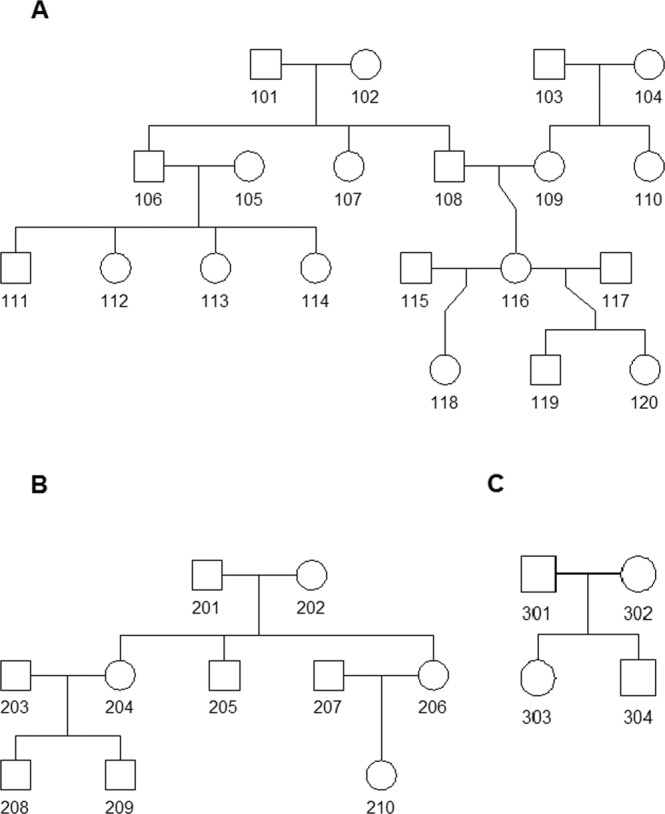
The structures of three simulated pedigrees.

We constructed a large pedigree (N = 20; Fig. 2A), a medium-size pedigree (N = 10; Fig. 2B), and a small pedigree (N = 4; Fig. 2C) as representatives to investigate the performance of our method. The genotype data of 18,210 markers on chromosome 22 were simulated from the HapMap phase 3 data. We first simulated the founders in the three pedigrees using the phased haplotypes from HapMap Yoruba in Ibdan, Nigeria (YRI), and Utah residents with Northern and Western European ancestry from the CEPH collection (CEU). For the first locus on this chromosome, we randomly sampled a YRI or CEU haplotype with probability given by the admixture rates (80%/20%). Moving along the chromosome, the recombination events were modeled with a Poisson distribution. Assuming at most one recombination event could occur between two adjacent loci, a recombination event was sampled with probability $(1-{e}^{-d\tau })$, where *d* is the genetic distance (in Morgans) and *τ* is the number of generations since admixture. We set *τ* = 8 for all founders. If a recombination event occurs, a new haplotype would be sampled. In the second step, we simulated the offspring given their parents’ haplotypes. The offspring inherits one chromosome from each of the parents. A crossover event in the parent was generated with probability $(1-{e}^{-d})$, i.e. *τ* = 1.

In practice, we do not know the true ancestral populations. To add more uncertainty in inferring local ancestry, we also inferred the local ancestries using HapMap phase 3 Luhya in Webuye, Kenya (LWK) and Toscani in Italy (TSI) as the reference panel. We used SABER+^13^ and HAPMIX^14^ to infer the local ancestries assuming all individuals are unrelated.

We estimated the allelic error rate *ε* from all markers on chromosome 22 using Eq. (8). Since a local ancestry block is often several cM long, to save computational time we could work on an ancestry block instead of on each single marker.

### Application: local ancestry inference in the cleveland family study

We applied FamANC on the African Americans from the Cleveland Family Study (CFS). The CFS is a family-based longitudinal study consisting of laboratory-diagnosed sleep apnea patients, their family members, and neighborhood control families, as described previously^16^. The de-identified genotype data were analyzed at Case Western Reserve University. The CFS study protocol was approved by the Partners Human Research Committee/IRB. The CFS includes 754 African Americans from 148 large families. Among those, 632 were genotyped with the Affymetrix 6.0 array and 122 were genotyped with the Illumina OmniExpress array. We merged the genotype data from the two different platforms and checked for Mendelian errors using PLINK^20^. Individuals with more than 5% Mendelian errors or SNPs with more than 10% Mendelian errors were removed. The remaining errors were set to be missing values. We phased the haplotypes in the CFS using BEAGLE software^21^ and inferred the local ancestries using HapMap phase 3 CEU and YRI as reference panels in SABER+ software, assuming all individuals are unrelated. We then applied FamANC on the SABER+ inferred local ancestries.

## Results

### Simulation

We used SABER+ and HAPMIX to infer the local ancestries on chromosome 22 in the three simulated families. The true dosage error rate for SABER+ and HAPMIX were similar (err = 0.011 vs 0.014). We divided the three families into 10 small nuclear families and estimated the allelic error rate *ε* from the observed Mendelian errors using Eq. (8) in those nuclear families. From Eq. (2), the estimated dosage error rate is 0.0096, which is consistent with the true inference error rate.

The ancestry inference error rates of SABER+ and HAPMIX for our simulated data are low. We modeled the probabilities of observing different numbers of individuals with ancestry inference errors at one locus in a family using a binomial distribution with inference error probability 0.01 (Supplementary Fig. 1). The probabilities of observing three or more individuals with ancestry error at the same locus were small in all three simulated pedigrees. Therefore, in a family with size no larger than 20, we do not have to search all possible ${X}_{t}\in Ped$. To save computation time we only considered a smaller set of *Ped* with at most two values different from the observed *Y*_*t*_.

By applying FamANC on the simulated data, we were able to correct local ancestry estimation errors at 195 loci per individual for SABER+ and at 90 loci per individual for HAPMIX (Table 1). The average dosage error rate of SABER+ was reduced from 0.011 to 0.0086, and the dosage error rate of HAPMIX was reduced from 0.014 to 0.0076.

**Table 1:** Performance of FamANC in simulated families.

	N	Software used in Step 1	Dosage error		Number of loci with errors	
			Step 1	Step 2	Step 1	Step 2
Family 1	20	SABER+	4456.3	3643	7217	3643
		HAPMIX	5543.5	3160	4723	3160
Family 2	10	SABER+	2305.4	1671	4623	1671
		HAPMIX	3993.7	2019	3451	2019
Family 3	4	SABER+	43.6	24	136	24
		HAPMIX	57.6	14	64	14
Total	34	SABER+	6805.3	5338	11976	5338
		HAPMIX	9594.8	5193	8238	5193

Step 1: local ancestry inferences using SABER+/HAPMIX while assuming individuals are unrelated. Step 2: Appling FamANC on top of the local ancestry results from Step 1.

### Local ancestries in CFS

We applied FamANC to the CFS African Americans. We checked Mendelian errors using PLINK^20^. No individual failed the 5% Mendelian filter threshold and no SNP failed the 10% Mendelian filter threshold. The genotypes with Mendelian errors across the genome were set as missing. We used SABER+ to estimate the local ancestries on phased chromosomes in the CFS assuming all individuals are unrelated. The local ancestry error rate, estimated from 50 nuclear families, was 0.0278, higher than that in the simulated data.

**Figure 3:**
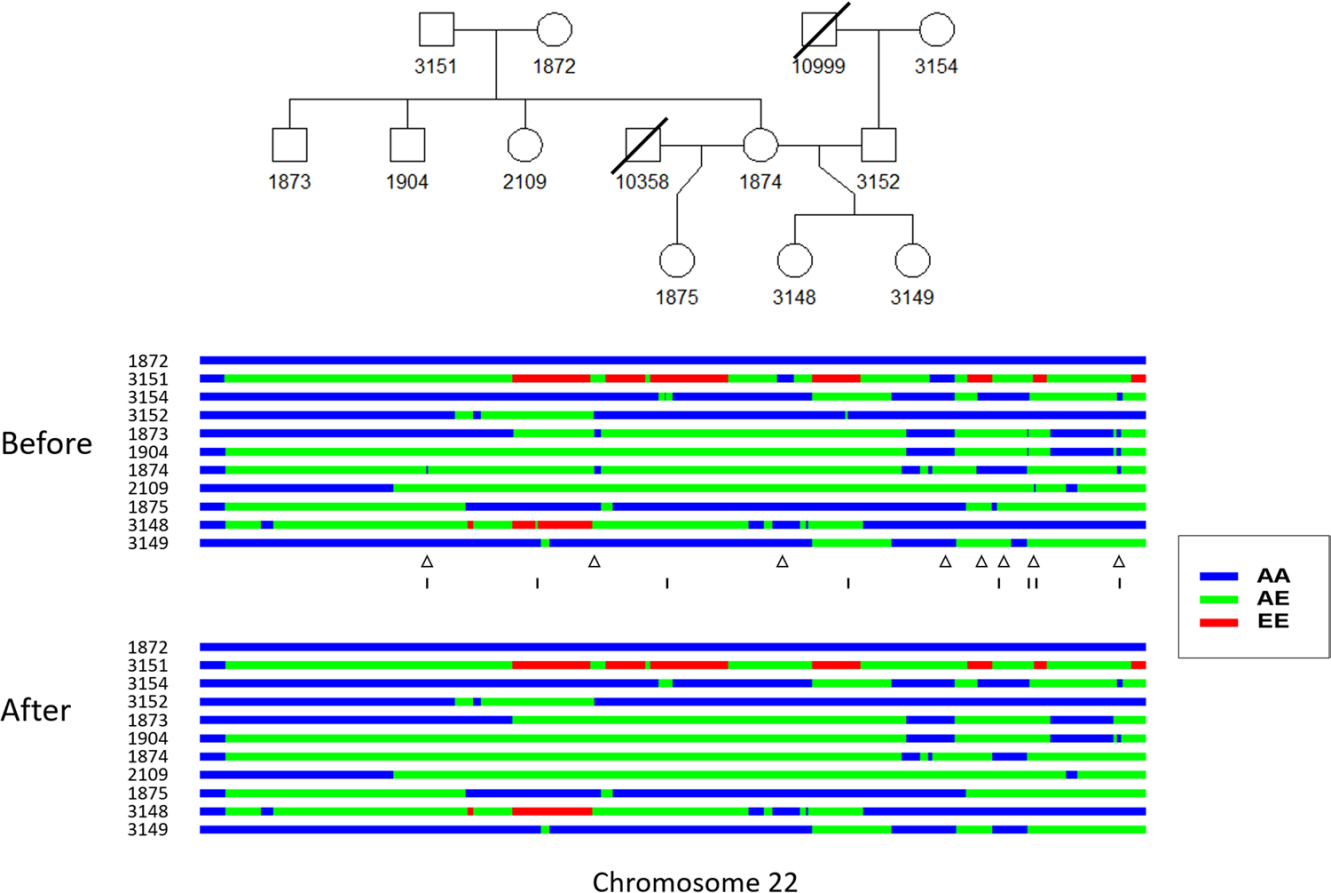
Estimated local ancestries on chromosome 22 in a family of 11 individuals from the CFS. Top: known pedigree structure. Middle: local ancestries estimated using a naïve approach that assumes that the individuals are unrelated. Bottom: local ancestries estimated by applying FamANC on the naïve estimates in combination with the known pedigree structure. Triangles indicate the regions with Mendelian errors found. Vertical bars indicate double crossovers corrected by FamANC.

For some pedigrees with missing first-generation genotype, we removed the first generation and divided them into smaller pedigrees (as seen in each dashed rectangle in Supplementary Fig. 2). A function for pedigree division is provided within the FamANC software. This resulted in 142 pedigrees with sizes ranging from 2 to 13. 124 individuals without any relatives collected in this dataset were further removed. The distribution of analyzed family size is presented in Supplementary Fig. 3A. We found the probability of observing three or more individuals with ancestry inference errors at the same locus for any family, given error rate = 0.03, to be small (Supplementary Fig. 3B). Therefore, to save computation time, we only searched for ${X}_{t}\in Ped$ with at most two values differing from the observed *Y*_*t*_ in a pedigree. Finally, we applied FamANC on the 142 families. Figure 3 shows the local ancestry estimates on chromosome 22 in an 11-individual family before and after applying FamANC. Our method was able to correct all identified Mendelian errors and most double crossovers. Having the local ancestries in the CFS with improved accuracy will be useful when using family-based admixture mapping to identify novel ancestry specific genetic risk factors for complex diseases such as sleep apnea. This approach may help to understand population differences of diseases and design personized treatment.

FamANC source code is available on GitHub (https://github.com/heming-wang/FamANC).

## Discussion

We have developed an efficient method, FamANC, that uses known pedigree structure to improve local ancestry inference in recently admixed populations, where local ancestry inference is first obtained by existing methods that (potentially falsely) assume study individuals are unrelated. Specifically, pedigree structure is used to identify and correct Mendelian errors and double crossovers. When applied on family data, this method reduces the systematic errors in local ancestry inference, thus having the potential to improve the performance of disease mapping studies and population genetics inference in recently admixed populations.

We have also provided a method to estimate the local ancestry inference error rate for existing software using the observed number of Mendelian errors in nuclear families while falsely assuming the individuals are unrelated. In this method, we assumed that in any small nuclear family only one member can have a local ancestry inference error at a locus. This assumption may be violated for an extremely large nuclear family with two parents and multiple children and lead to underestimation of the error rate.

We estimated a higher local ancestry inference error rate in real data than in simulated data. This could be due to genotyping errors in the real data. However, it is also possible that our simulation strategy may only reflect an ideal mixture of ancestral haplotype segments, which may not represent the complex admixture process of ancestral populations in evolutionary history. This simulation method has been commonly used in many genetic studies, so this possibility raises a concern about the performance of many local ancestry inference methods. Developing mathematical models that mimic a complex and historically accurate admixture history is a topic for future research.

Our method has some limitations. FamANC performs well when the local ancestries inferred in the sample at the first step, assuming independence, are estimated with sufficient accuracy (err < 0.1). It may not be suitable for poorly inferred data. At a given locus, our method works by detecting either a Mendelian or a cross-over error, and correcting it using local ancestry inference from neighboring loci, namely ${X}_{t-1},{X}_{t+1}$. Doing so, we assume that the inferred local ancestries in these positions are correct, i.e. ${X}_{t-1}={Y}_{t-1}$ and ${X}_{t+1}={Y}_{t+1}$. Clearly, this is not always true. For example, in our simulation we observed a type of local ancestry inference error introduced by shifting recombination points (Supplementary Fig. 4), which results in incorrect inference along an interval, and this may overlap multiple loci. However, none of the existing methods appropriately handles such an error. Detecting and correcting for such a shifting recombination point error is a topic for future research. Other potential improvements to FamANC can also be gained by incorporating more markers around each locus for error correction, and by using genotypes. In this study, we only evaluated the performance of FamANC on local ancestries inferred by SABER+ and HAPMIX, which were commonly used in two-way admixed population and showed high accuracy^22^. The performance of FamANC when used following other recently released ancestry inference software such as RFMix^23^ should be evaluated in the future.

In summary, we developed a novel method, FamANC, which can improve the accuracy of local ancestry inference in large pedigrees and will benefit future family-based genetic studies.

## Supplementary information

Supplementary Information.
