# Supplementary material for: Local Ancestry Inference in Large Pedigrees
Source: Sci Rep. 2020 Jan 13;10:189. doi: 10.1038/s41598-019-57039-w (PMC6957497; doi:10.1038/s41598-019-57039-w)
Supplement: Supplementary file 1 — Supplementary Information. [file 41598_2019_57039_MOESM1_ESM.docx]

**Supplementary Information**

**Local Ancestry Inference in Large Pedigrees**

**Heming Wang^1,2,3,*^, Tamar Sofer^1,2^, Xiang Zhang^4^, Robert C. Elston^3^, Susan Redline^1,5^, Xiaofeng Zhu^3^**

^1^ Division of Sleep and Circadian Disorders, Brigham and Women’s Hospital and Harvard Medical School, Boston, MA, USA

^2^ Program in Medical and Population Genetics, Broad Institute, Cambridge, MA, USA

^3^ Department of Population and Quantitative Health Sciences, Case Western Reserve University, Cleveland, OH, USA

^4^ College of Information Sciences and Technology, Pennsylvania State University, PA, USA

^5^ Department of Sleep Medicine, Beth Israel Deaconess Medical Center, Boston, MA, USA

**Supplementary Figure 1.** The probability of numbers of individuals with ancestry errors at the same locus in three simulated pedigree with error rate 0.01.

**
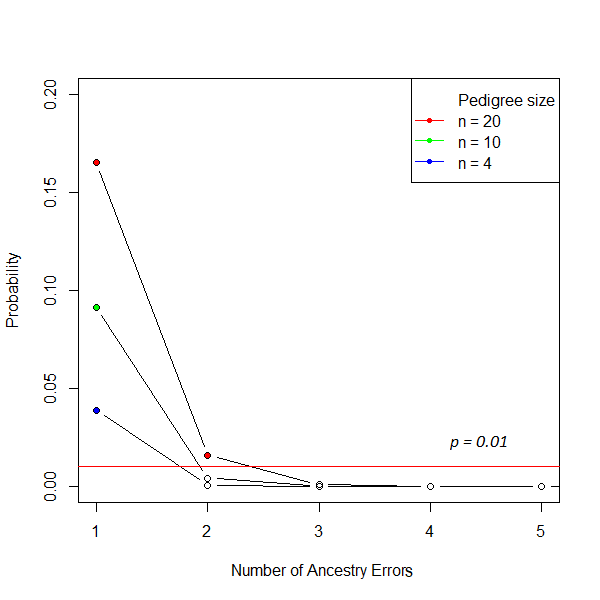
**

**Supplementary Figure 2.** Dividing a large pedigree into smaller informative pedigrees.

**
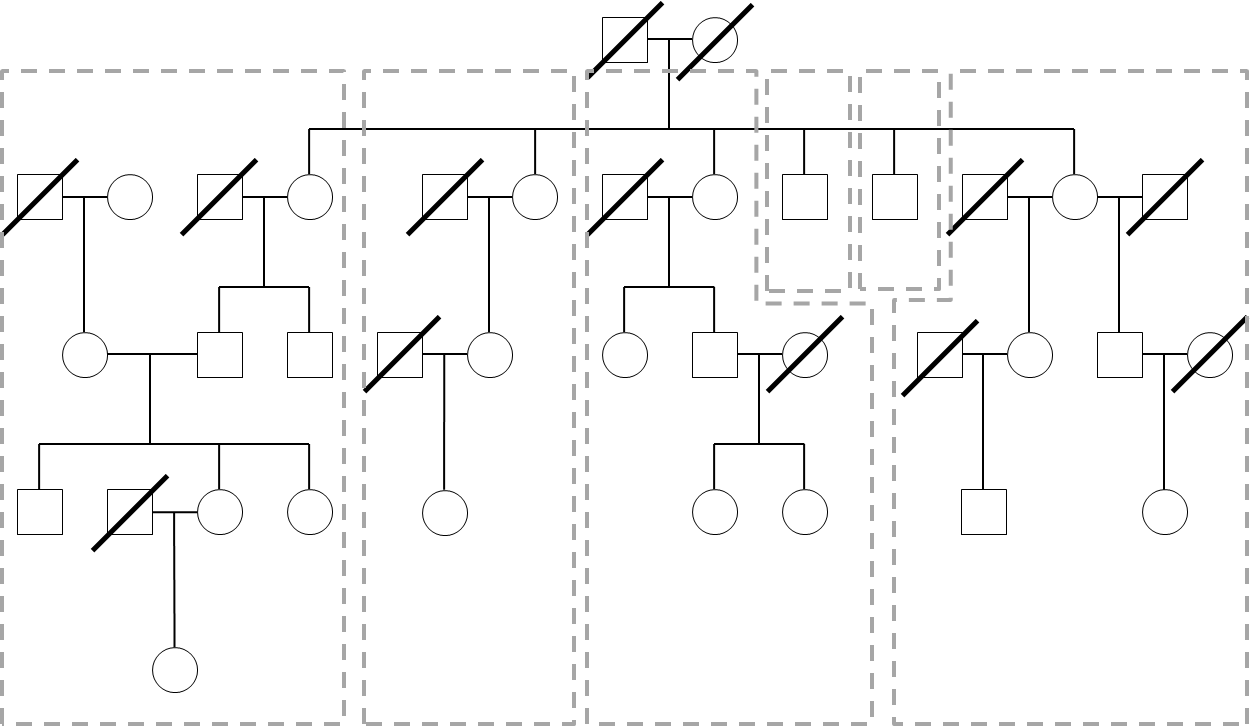
**

**Supplementary Figure 3.** Pedigree characteristics in the CFS. (A) Histogram of analyzed pedigree sizes. (B) The probability of numbers of individuals with ancestry errors at the same locus in pedigrees with error rate of 0.03.

**
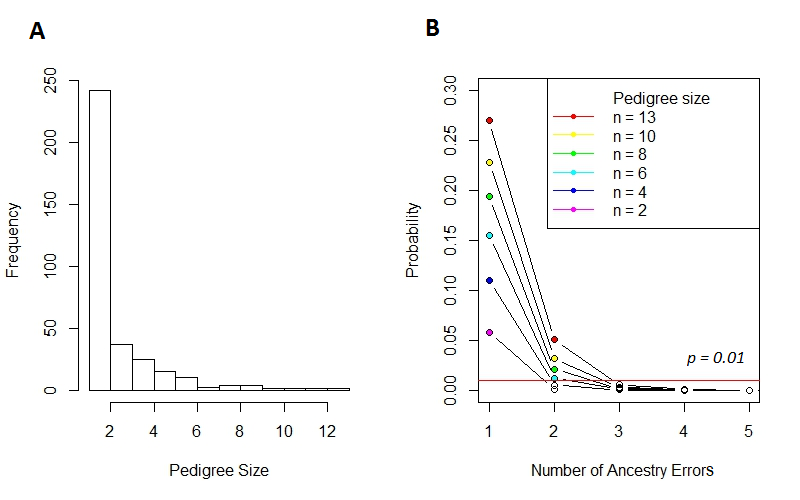
**

**Supplementary Figure 4.** An example of local ancestry inference error that cannot be corrected by FamANC. In this family of three individuals (Father, Mother, Child), the recombination point between the inferred local ancestries in Mother shifts to the right of the true recombination point. The local ancestry inference error in the bolded rectangle cannot be identified and corrected using FamANC.


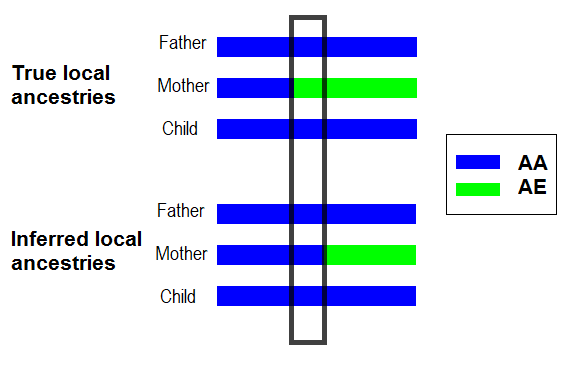


**Supplementary Table 1.** The probability of an inferred local ancestry $Y_{i,t}$ given the true local ancestry $X_{i,t}$ at the *t^th^* locus.

| $X_{i,t}$ | $P\left( Y_{i,t}=0 \vert X_{i,t} \right)$ | $P\left( Y_{i,t}=1 \vert X_{i,t} \right)$ | $P\left( Y_{i,t}=2 \vert X_{i,t} \right)$ | $P(X_{i,t})$ |
| --- | --- | --- | --- | --- |
| $0$ | ${(1-\varepsilon)}^{2}$ | $2\varepsilon(1-\varepsilon)$ | $\varepsilon^{2}$ | $\lambda^{2}$ |
| $1$ | $\varepsilon(1-\varepsilon)$ | $\varepsilon^{2}+{(1-\varepsilon)}^{2}$ | $\varepsilon(1-\varepsilon)$ | $2\lambda(1-\lambda)$ |
| $2$ | $\varepsilon^{2}$ | $2\varepsilon(1-\varepsilon)$ | ${(1-\varepsilon)}^{2}$ | ${(1-\lambda)}^{2}$ |

**Appendix 1.** Estimating local ancestry inference errors from Mendelian inconsistency.

We use the African American population to illustrate the mathematical deduction. Assume, at a single marker, A is the African allele and E is European allele. In a nuclear family with two parents and *m* children, we denote F, M, and C_j_ as the true local ancestries of the father, mother, and the j^th^ child; and F^obs^, M^obs^, and C_j_^obs^ as the inferred local ancestries of the father, mother, and the j^th^ child with errors. The probabilities of observing a child’s local ancestry given his/her parents’ local ancestries are presented in Supplementary Table 2.

**Supplementary Table 2.** The probability of the j^th^ child’s local ancestry given his/her parents’ local ancestries.

|  | C_j_ | | |
| --- | --- | --- | --- |
| (F, M) | AA | AE | EE |
| (AA,AA) | 1 | 0 | 0 |
| (AA,AE) | ½ | ½ | 0 |
| (AA,EE) | 0 | 1 | 0 |
| (AE,AA) | ½ | ½ | 0 |
| (AE,AE) | ¼ | ½ | ¼ |
| (AE,EE) | 0 | ½ | ½ |
| (EE,AA) | 0 | 1 | 0 |
| (EE,AE) | 0 | ½ | ½ |
| (EE,EE) | 0 | 0 | 1 |

Let ME be the set of local ancestries in a family with Mendelian errors and $\bar{\mathrm{ME}}$ be the set of local ancestries in a family without Mendelian errors. We first consider the patterns in which only the father’s local ancestry is in error. From Supplementary Table 3, we can calculate the probability of the father’s local ancestry being in error but not leading to a Mendelian error in this family.

**Supplementary Table 3.** The probability of the father’s local ancestry being in error but not leading to Mendelian errors in a family of 2 parents and *m* children.

| F | F^obs^ | M | P(F^obs^\|F) | P(F,M) | $P(\bar{\mathrm{ME}}\vert F^{\mathrm{obs}},F,M)$ |
| --- | --- | --- | --- | --- | --- |
| AA | AE | AA | 2ε(1-ε) | λ^4^ | 1 |
|  |  | AE | 2ε(1-ε) | 2λ^3^(1-λ) | 1 |
|  |  | EE | 2ε(1-ε) | λ^2^(1-λ)^2^ | 1 |
|  | EE | AA | ε^2^ | λ^4^ | 0 |
|  |  | AE | ε^2^ | 2λ^3^(1-λ) | (½)^m^ |
|  |  | EE | ε^2^ | λ^2^(1-λ)^2^ | 0 |
| AE | AA | AA | ε(1-ε) | 2λ^3^(1-λ) | (½)^m^ |
|  |  | AE | ε(1-ε) | 4λ^2^(1-λ)^2^ | (¾)^m^ |
|  |  | EE | ε(1-ε) | 2λ(1-λ)^3^ | (½)^m^ |
|  | EE | AA | ε(1-ε) | 2λ^3^(1-λ) | (½)^m^ |
|  |  | AE | ε(1-ε) | 4λ^2^(1-λ)^2^ | (¾)^m^ |
|  |  | EE | ε(1-ε) | 2λ(1-λ)^3^ | (½)^m^ |
| EE | AA | AA | ε^2^ | λ^2^(1-λ)^2^ | 0 |
|  |  | AE | ε^2^ | 2λ(1-λ)^3^ | (½)^m^ |
|  |  | EE | ε^2^ | (1-λ)^4^ | 0 |
|  | EE | AA | 2ε(1-ε) | λ^2^(1-λ)^2^ | 1 |
|  |  | AE | 2ε(1-ε) | 2λ(1-λ)^3^ | 1 |
|  |  | EE | 2ε(1-ε) | (1-λ)^4^ | 1 |

$P\left( \mathrm{the}\mathrm{father}^{'}s local ancestry is in error but no Mendelian error in this family \right)=P(\bar{\mathrm{ME}}|F^{\mathrm{obs}},F,M)\times P(F,M)\times P(F^{\mathrm{obs}}|F) =2\varepsilon\left( 1-\varepsilon\right)\left\{ \lambda^{2}+\left( 1-\lambda\right)^{2}+\left( \frac{1}{2} \right)^{m-1}\lambda\left( 1-\lambda\right)+4\lambda^{2}\left( 1-\lambda\right)^{2}\left[ \left( \frac{3}{4} \right)^{m}-\left( \frac{1}{2} \right)^{m} \right] \right\}+\varepsilon^{2}\lambda\left( 1-\lambda\right)\left[ \lambda^{2}+\left( 1-\lambda\right)^{2} \right]\left( \frac{1}{2} \right)^{m-1}$.

The probability of the mother’s local ancestry being in error but not leading to Mendelian errors is analogous.

The patterns of the j^th^ child’s local ancestry being in error are shown in Supplementary table 4.

**Supplementary Table 4.** The probability of the j^th^ child’s local ancestry is being in error but not leading to Mendelian errors in a family of 2 parents and *m* children.

| F | M | C_j_ | $C_{j}$^obs^ | P($C_{j}$^obs^\|$C_{j}$) | P(M,F) | P($C_{j}$\|M,F) | $P(\bar{\mathrm{ME}}\vert{C_{j}}^{\mathrm{obs}},C_{j},F,M)$ |
| --- | --- | --- | --- | --- | --- | --- | --- |
| AA | AA | AA | AE | 2ε(1-ε) | λ^4^ | 1 | 0 |
|  |  |  | EE | ε^2^ | λ^4^ | 1 | 0 |
|  | AE | AA | AE | 2ε(1-ε) | 2λ^3^(1-λ) | ½ | 1 |
|  |  |  | EE | ε^2^ | 2λ^3^(1-λ) | ½ | 0 |
|  |  | AE | AA | ε(1-ε) | 2λ^3^(1-λ) | ½ | 1 |
|  |  |  | EE | ε(1-ε) | 2λ^3^(1-λ) | ½ | 0 |
|  | EE | AE | AA | ε(1-ε) | λ^2^(1-λ)^2^ | 1 | 0 |
|  |  |  | EE | ε(1-ε) | λ^2^(1-λ)^2^ | 1 | 0 |
| AE | AA | AA | AE | 2ε(1-ε) | 2λ^3^(1-λ) | ½ | 1 |
|  |  |  | EE | ε^2^ | 2λ^3^(1-λ) | ½ | 0 |
|  |  | AE | AA | ε(1-ε) | 2λ^3^(1-λ) | ½ | 1 |
|  |  |  | EE | ε(1-ε) | 2λ^3^(1-λ) | ½ | 0 |
|  | AE | AA | AE | 2ε(1-ε) | 4λ^2^(1-λ)^2^ | ¼ | 1 |
|  |  |  | EE | ε^2^ | 4λ^2^(1-λ)^2^ | ¼ | 1 |
|  |  | AE | AA | ε(1-ε) | 4λ^2^(1-λ)^2^ | ½ | 1 |
|  |  |  | EE | ε(1-ε) | 4λ^2^(1-λ)^2^ | ½ | 1 |
|  |  | EE | AA | ε^2^ | 4λ^2^(1-λ)^2^ | ¼ | 1 |
|  |  |  | AE | 2ε(1-ε) | 4λ^2^(1-λ)^2^ | ¼ | 1 |
|  | EE | AE | AA | ε(1-ε) | 2λ(1-λ)^3^ | ½ | 0 |
|  |  |  | EE | ε(1-ε) | 2λ(1-λ)^3^ | ½ | 1 |
|  |  | EE | AA | ε^2^ | 2λ(1-λ)^3^ | ½ | 0 |
|  |  |  | AE | 2ε(1-ε) | 2λ(1-λ)^3^ | ½ | 1 |
| EE | AA | AE | AA | ε(1-ε) | λ^2^(1-λ)^2^ | 1 | 0 |
|  |  |  | EE | ε(1-ε) | λ^2^(1-λ)^2^ | 1 | 0 |
|  | AE | AE | AA | ε(1-ε) | 2λ(1-λ)^3^ | ½ | 0 |
|  |  |  | EE | ε(1-ε) | 2λ(1-λ)^3^ | ½ | 1 |
|  |  | EE | AA | ε^2^ | 2λ(1-λ)^3^ | ½ | 0 |
|  |  |  | AE | 2ε(1-ε) | 2λ(1-λ)^3^ | ½ | 1 |
|  | EE | EE | AA | ε^2^ | (1-λ)^4^ | 1 | 0 |
|  |  |  | AE | 2ε(1-ε) | (1-λ)^4^ | 1 | 0 |

$P\left( \mathrm{the}j^{th} child's local ancestry is in error but no Mendelian error in this family \right)=P(\bar{\mathrm{ME}}|{C_{j}}^{\mathrm{obs}},C_{j},F,M)\times P(C_{j}|F,M)\times P(F,M)\times P({C_{j}}^{\mathrm{obs}}|C_{j})=3\varepsilon\left( 1-\varepsilon\right)\lambda\left( 1-\lambda\right)\left[ 3-2\lambda\left( 1-\lambda\right) \right]+2\varepsilon^{2}\lambda^{2}\left( 1-\lambda\right)^{2}$

This probability needs to be multiplied by *m,* considering all *m* children in the nuclear family.

Let $N_{ME}$ be the number of Mendelian errors in that nuclear family. $N_{ME}$ can be calculated as the total local ancestry error minus the number of local ancestry errors not leading to Mendelian inconsistency.;

$N_{ME}=(2+m) E-2P\left( \bar{\mathrm{ME}},\mathrm{LE}_{F} \right)-mP\left( \bar{\mathrm{ME}},\mathrm{LE}_{C_{j}} \right)$.

From Equation (2), the total local ancestry error is the error rate E multiplied by (2+m), the number of individuals in the family:

$\left( 2+m \right)E=(2+m)\left\{ 2\varepsilon\left( 1-\varepsilon\right)+\varepsilon^{2}\left[ \lambda^{2}+{(1-\lambda)}^{2} \right] \right\}$.

After some algebra,

$$N_{ME}=$$

$$\varepsilon^{2}\left\{ 2-m+\left[ 4m-12+\left( \frac{1}{2} \right)^{m-2} \right]\lambda\left( 1-\lambda\right)-\left[ 6m-16\left( \frac{3}{4} \right)^{m}+\left( \frac{1}{2} \right)^{m-3} \right]\lambda^{2}{(1-\lambda)}^{2} \right\}$$

$+\varepsilon\left\{ 2m+\left[ 8-6m-\left( \frac{1}{2} \right)^{m-3} \right]\lambda\left( 1-\lambda\right)+\left[ 4m-16\left( \frac{3}{4} \right)^{m}+\left( \frac{1}{2} \right)^{m-4} \right]\lambda^{2}\left( 1-\lambda\right)^{2} \right\}$. (8)

If one parent is missing (e.g. the mother), the probability that she contributes the African allele is estimated from the admixture rate of the African ancestry λ. The probability of a child’s local ancestry given the father’s local ancestry is presented in Supplementary Table 5.

**Supplementary Table 5.** The probability of the j^th^ child’s local ancestry given the father’s local ancestry when the mother is missing.

|  | Cj | | |
| --- | --- | --- | --- |
| F | EE | AE | AA |
| EE | 1-λ | λ | 0 |
| AE | ½(1-λ) | ½ | ½λ |
| AA | 0 | 1-λ | Λ |

Similarly, we use Supplementary Table 6 to summarize the patterns of local ancestry errors in the parent but not leading to Mendelian inconsistency in a family.

**Supplementary Table 6.** The probability of the father’s local ancestry is being in error but not leading to Mendelian errors in a family of 1 parent and *m* children.

| F | F^obs^ | P(F^obs^\|F) | P(F) | $P(\bar{\mathrm{ME}}\vert F^{\mathrm{obs}},F)$ |
| --- | --- | --- | --- | --- |
| AA | AE | 2ε(1-ε) | λ^2^ | 1 |
|  | EE | ε^2^ | λ^2^ | (1-λ)^m^ |
| AE | AA | ε(1-ε) | 2λ(1-λ) | [1-½(1-λ)]^m^ |
|  | EE | ε(1-ε) | 2λ(1-λ) | (1-½λ)^m^ |
| EE | AA | ε^2^ | (1-λ)^2^ | λ^m^ |
|  | EE | 2ε(1-ε) | (1-λ)^2^ | 1 |

$P\left( the father's local ancestry is in error but no Mendelian error in this family \right)=2\varepsilon\left( 1-\varepsilon\right)\lambda^{2}+\varepsilon^{2}\lambda^{2}\left( 1-\lambda\right)^{m}+\varepsilon\left( 1-\varepsilon\right)\cdot2\lambda\left( 1-\lambda\right)\left[ 1-\frac{1}{2}\left( 1-\lambda\right) \right]^{m}+\varepsilon\left( 1-\varepsilon\right)\cdot2\lambda\left( 1-\lambda\right)\left[ 1-\frac{1}{2}\lambda\right]^{m}+\varepsilon^{2}\left( 1-\lambda\right)^{2}\lambda^{m}+2\varepsilon\left( 1-\varepsilon\right)\left( 1-\lambda\right)^{2}=2\varepsilon\left( 1-\varepsilon\right)\left[ \lambda^{2}+\left( 1-\lambda\right)^{2} \right]+2\varepsilon\left( 1-\varepsilon\right)\lambda\left( 1-\lambda\right)\left\{ {\left( \frac{1}{2}+\frac{1}{2}\lambda\right)^{m}+\left( 1-\frac{1}{2}\lambda\right)}^{m} \right\}+\varepsilon^{2}\lambda^{2}\left( 1-\lambda\right)^{2}\left[ \lambda^{m-2}+{(1-\lambda)}^{m-2} \right]$.

Supplementary Table 7 summarizes the patterns of local ancestry errors in one child that lead to Mendelian errors, because it is easier to list all Mendelian errors than the cases of no Mendelian errors.

**Supplementary Table 7.** The probability of the j^th^ child’s local ancestry being in error leading to Mendelian errors in a family of 1 parent and *m* children.

| F | C_j_ | $C_{j}$^obs^ | P($C_{j}$^obs^\|$C_{j}$) | P(F) | P(C_j_\|F) |
| --- | --- | --- | --- | --- | --- |
| AA | AA | EE | ε^2^ | λ^2^ | λ |
|  | AE | EE | ε(1-ε) | λ^2^ | 1-λ |
| EE | AE | AA | ε(1-ε) | (1-λ)^2^ | λ |
|  | EE | AA | ε^2^ | (1-λ)^2^ | 1-λ |

$P\left( the jth \mathrm{child}^{'}s local ancestry is in error leading to Mendelian error in this family \right)=\varepsilon^{2}\left[ \lambda^{3}+\left( 1-\lambda\right)^{3} \right]+\varepsilon\left( 1-\varepsilon\right)\lambda\left( 1-\lambda\right).$

After some algebra, the number of Mendelian errors in such a family is

$$N_{ME}=2\varepsilon\left( 1-\varepsilon\right)\lambda\left( 1-\lambda\right)\left\{ 2-\left( 1-\frac{1}{2}\lambda\right)^{m}-\left( \frac{1}{2}+\frac{1}{2}\lambda\right)^{m}+\frac{1}{2}m \right\}$$

$+\varepsilon^{2}\left\{ \lambda\left[ 1+\lambda-\left( 1-\lambda\right)^{m} \right]+\left( 1-\lambda\right)^{2}\left( 2-\lambda+\lambda^{m} \right) \right\}$ . (9)

We can estimate $\varepsilon$ by solving Equations (8) and (9).
